# Pituitary Metastatic Composite Tumors: A Case Report with Next-Generation Sequencing and Review of the Literature

**DOI:** 10.1155/2020/5073236

**Published:** 2020-07-21

**Authors:** Matthew Helton, Muhammad Abu-Rmaileh, Kevin Thomas, Murat Gokden, Alissa Kanaan, Analiz Rodriguez

**Affiliations:** ^1^Department of Neurosurgery, University of Arkansas for Medical Sciences, Little Rock, AR 72205, USA; ^2^Division of Neuropathology, Department of Pathology, University of Arkansas for Medical Sciences, Little Rock, AR 72205, USA; ^3^Department of Otolaryngology, University of Arkansas for Medical Sciences, Little Rock, AR 72205, USA

## Abstract

**Background:**

While pituitary tumors are well understood, little research has been done on metastasis from primary tumors into pituitary adenomas, also known as composite tumors. Because only 34 cases of composite tumors have been reported to date, we hope to better characterize these tumors by reviewing cases reported in the literature and reviewed our own documented case, which includes next-generation sequencing. *Case Presentation*. A 74-year-old man presented to the emergency department with left vision loss for 3 months. He had a history of colon cancer treated with colectomy and clear cell renal carcinoma treated with left nephrectomy. A preoperative MRI demonstrated growth of a peripherally enhancing, centrally necrotic mass with sellar expansion measuring 5.7 × 3.1 × 3.0 cm. Given these findings, an endoscopic endonasal transsphenoidal resection was performed. Histological assessment revealed a composite tumor: one neoplasm was a nonfunctioning pituitary adenoma, and another neoplasm was a clear cell carcinoma. Next-generation sequencing demonstrated that the tumors shared mutations in *VHL* and *Notch*2. The patient died 2 months later from systemic metastatic cancer.

**Conclusion:**

From our literature review, most metastatic lesions in these composite tumors originated from neoplasms of the lung and kidney. Approximately 63% patients presented with ophthalmoplegia as the initial symptom while 23% displayed hormonal abnormalities. Postoperative mortality had a median of 3.5 months. In our patient, the presence of *VHL* and *Notch*2 mutations in both tumors highlights the possibility of using next-generation sequencing to help identify therapeutic targets even in complex composite neoplasms.

## 1. Background

In general, pituitary tumors range in incidence from 0.038% to 0.12% with pituitary adenomas being the most common [1]. In neurosurgical series, tumors of the pituitary, meaning pituitary adenoma or carcinoma, represent 16.5% of all neoplasms, with only a small minority of these neoplasms representing pituitary carcinoma [2]. Metastases to the pituitary gland have been reported to occur in approximately 2% of patients with diagnosed pituitary masses [3]. In autopsy series, pituitary metastases have been reported to be as high as 27% among patients with malignant tumors [4]. As reported in the autopsy series, only 7% of pituitary metastases are reported as symptomatic, which leads to clinical diagnosis, with reported symptoms of diabetes insipidus, ophthalmoplegia, headache, visual field defects, and anterior pituitary dysfunction. Primary malignant tumors of the breast and lung are the most common metastatic lesions found in the pituitary [5].

While metastatic lesions to the pituitary gland or sella turcica have been well described in the literature, a lesser known entity is a composite tumor involving metastases and pituitary adenomas. Only 34 cases have been reported in the literature to date. We aim to better characterize these rare lesions by reviewing reported cases and describing a recently observed case, which includes next-generation sequencing, at our institution.

## 2. Case Report

**Figure 1:**
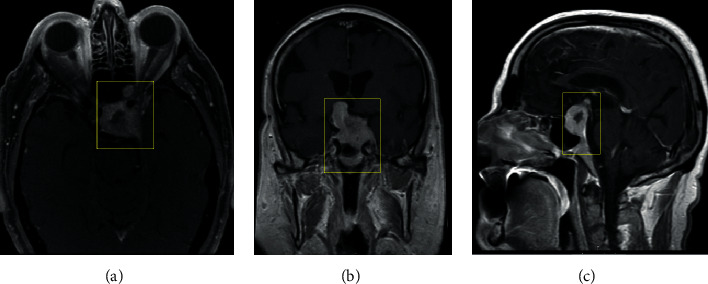
Radiographic imaging, on initial workup of the heterogeneously enhancing eccentric. T1-weighted sequence magnetic resonance imaging with contrast presented in axial, coronal, and sagittal planes, respectively. The mass invades the left cavernous sinus and orbit with envelopment of the left internal carotid artery (a). In the suprasellar region, the mass extends into the right hypothalamus (b) with mass effect on the third ventricle (c). The mass measures 4.3 x 3.3 x 3.7 cm with a calculated volume of 17.11 mL using 3D modelling. Boxes highlight the tumor of interest.

A 74-year-old man was referred to our institution's neurosurgery and otolaryngology department after an outside ophthalmologist ordered an MRI revealing a sellar mass. The patient had a history of left vision loss for the past 3 months prior to his visit. He had a distant history of colon cancer treated with colectomy and was diagnosed 4 years ago with clear cell renal carcinoma that was subsequently treated with left nephrectomy. He was found to have a secondary lung lesion two years prior but did not pursue treatment. An MRI of the brain performed one month previously at an outside hospital displayed a sellar mass with suprasellar extension (4.6 × 3.4 cm) into the orbital apex and cavernous sinus on the left side (Figure 1).

The left eye had lost vision except for a preserved small portion of the upper nasal field. Serum values of prolactin were marginally elevated at 17.86. Given these findings, an endoscopic endonasal transsphenoidal resection of the lesion was recommended.

**Figure 2:**
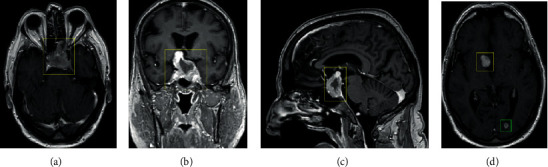
Radiographic imaging of tumor progression preoperatively. T1-weighted sequence magnetic resonance imaging with contrast presented in axial, coronal, sagittal, and axial planes, respectively. Imaging demonstrates growth of the previously described mass with further encasement of the left cavernous internal carotid artery (a) with superior extension (b) with further effacement of the third ventricle (c). A subcentimeter-enhancing, spherical, paraventricular, left occipital lesion is apparent (d). The sellar mass now measures 5.7 x 3.8 x 4.4 cm with a total estimated volume of 22.7 mL. Yellow boxes highlight the tumor of interest. Green box highlights an occipital lesion.

**Figure 3:**
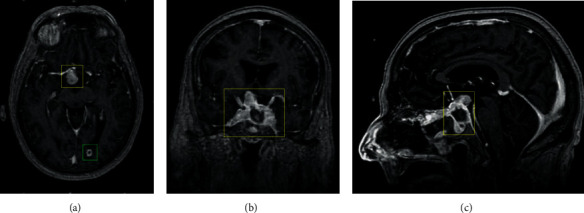
Radiographic imaging of tumor progression postoperative. T1-weighted sequence magnetic resonance imaging with double contrast presented in axial, coronal, and sagittal planes, respectively. The mass has undergone subtotal resection (a–c). The left occipital lesion is stable (a). The suprasellar lesion measures 2.7 x 1.4 x 2.0 cm with total volume calculated at 4.6 mL. Yellow boxes highlight the tumor of interest. Green box highlights occipital lesion.

A preoperative MRI with and without contrast demonstrated growth to a 5.7 × 3.1 × 3.0 cm peripherally enhancing, centrally necrotic mass with sellar expansion (Figure 2). The patient underwent surgery one month later after referral. Tumor volume decreased from 22.70 mL preoperatively to 4.59 mL postoperatively (Figure 3). During postoperative imaging, a new intracranial lesion was noticed in the left occipital lobe (Figure 2(d)).

**Figure 4:**
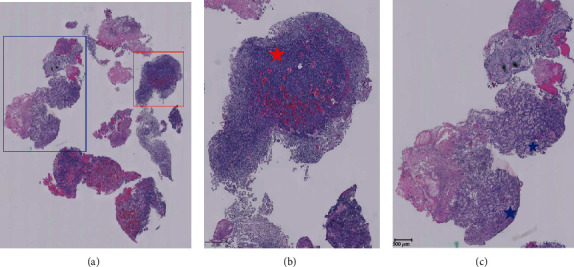
Histopathologic stains of the pituitary mass. (a) H and E stain of the pituitary lesion. (b) Magnified image of red box in a showing evidence of pituitary adenoma which is surrounded by clear cells that resemble RCC but without the nuclear changes. (c) Magnified image of blue box in (a) showing clear cytoplasm arranged in nests with severe nuclear atypia consistent with WHO/ISUP grade 3 RCC.

**Figure 5:**
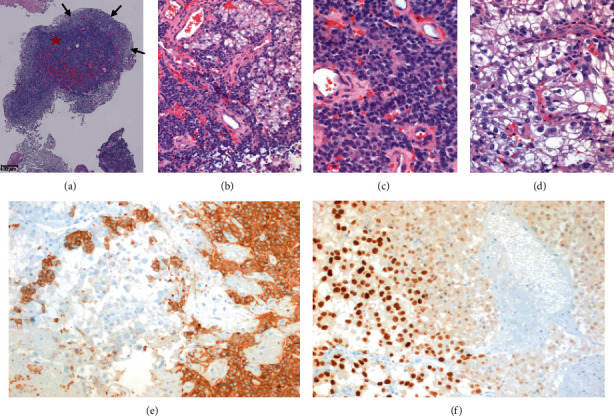
(a) Admixed pituitary adenoma (star) and the surrounding metastatic carcinoma (arrows) in one of the fragments. (b) Juxtaposition of pituitary adenoma (lower left) and metastatic renal cell carcinoma (upper right). (c) Pituitary adenoma shows sheets of cytologically bland, monotonous cells. (d) Renal cell carcinoma shows large atypical cells with prominent nucleoli and clear cytoplasms. (e) Synaptophysin is positive in the pituitary adenoma and negative in the renal cell carcinoma. PAX-8 shows nuclear positivity in the renal cell carcinoma, while the pituitary adenoma is negative. (a–e) Hematoxylin-eosin. (d, e) Immunohistochemistry. Original magnifications: (a) 20x; (b, e, f) 200x; and (c, d) 400x.

Microscopic examination showed two neoplasms that were present in separate fragments, as well as juxtaposed or intimately admixed in some others. One component was a pituitary adenoma with no unusual or atypical features. It had a low Ki-67 proliferation index and was immunohistochemically positive for cytokeratins (AE1/AE3 and CK 8/18) and synaptophysin. Prolactin was weakly positive in some areas, along with scattered, luteinizing hormone- (LH-) positive cells. Growth hormone, adrenocorticotrophic hormone, follicle-stimulating hormone, and luteinizing hormone stains were negative. The other neoplastic component was composed of large, atypical cells with clear cytoplasms and prominent nucleoli. Areas of necrosis were associated with this component. It was also positive for cytokeratins (AE1/AE3 and CK 8/18), PAX-8, and renal cell carcinoma marker (RCC-Ma), coexpressed vimentin. It was negative for synaptophysin, pituitary hormones, CK7 and CK20, prostate-specific antigen, and thyroid transcription factor-1. The microscopic findings and immunophenotype indicated a metastatic clear cell renal cell carcinoma to a pituitary adenoma (Figures 4 and 5). On a digital pathology analysis, the pituitary adenoma to renal cell carcinoma ratio was 2.70.

Genetic analysis of the pituitary adenoma component revealed *VHL* p.P86S missensense variant with a loss of function (variant allele frequency (VAF) of 4.8%) with additional *FAT1*, *HNF1A*, and *NOTCH2* copy number loss. Gene analysis of the renal cell carcinoma component revealed *PBRM1* p.K651^∗^ stop gain (VAF 25.1%), *VHL* p.P86S missense variant with a loss of function (VAF 24.7%), *NF1* p.N510Y splice region variant (VAF 5.7%), STAG2 p.R216^∗^ stop gain with loss of function (VAF 3.9%), and *NOTCH2* copy number loss. Therefore, overlapping mutations in *VHL* and *NOTCH2* were present in both components of the tumor. Unfortunately, next-generation sequencing was done on archival specimens after the patient expired and germ cell tissue was not available to affirm whether the patient had Von Hippel Lindau disease.

Following surgery, the patient had an uncomplicated postoperative course and reported visual improvement.

The patient returned to the hospital on postoperative day 10 for stereotactic radiosurgery to the newly diagnosed L occipital metastatic lesion. Stereotactic radiosurgery was not able to be performed on the suprasellar tumor as there was not enough separation from the optic nerves.

On the initial postoperative clinic visit, the patient stated that he experienced an improvement in vision; however, the patient was found to have systemic metastatic cancer including significant tumor burden on his lungs. The patient refused further care for the systemic metastatic cancer. The patient died two months later.

## 3. Methods

After obtaining consent from our institutional review board (IRB #239292), we reviewed one observed case of metastatic disease and pituitary adenoma coexisting in the sella turcica and suprasellar space and performed next-generation sequencing on the composite tumor.

### 3.1. Genomics Technique

DNA and RNA sequencing was performed on the patient's tumor specimens, using the xT Laboratory Developed Test at Tempus' Clinical Laboratory Improvement Amendments/College of American Pathologists-accredited laboratory in Chicago, IL. Tumor DNA was extracted from tumor tissue sections with tumor cellularity higher than 20% and proteinase K digested. Total nucleic acid extraction was performed with a Chemagic360 instrument using a source-specific magnetic bead protocol. Total nucleic acid was utilized for DNA library construction, while RNA was further purified by DNaseI digestion and magnetic bead purification. The nucleic acid was quantified by a Quant-iT picogreen dsDNA reagent Kit or Quant-iT Ribogreen RNA Kit (Life Technologies), and quality is confirmed using a LabChip GX Touch HT Genomic DNA Reagent Kit or LabChip RNA High HT Pico Sensitivity Reagent Kit (PerkinElmer).

For the DNA library construction, one hundred nanograms of DNA for each tumor and normal sample was mechanically sheared to an average size of 200 base pairs using a Covaris ultrasonicator. The libraries were prepared using the KAPA Hyper Prep Kit. Briefly, DNA underwent enzymatic end-repair and A-tailing, followed by adapter ligation, bead-based size selection, and PCR. After library preparation, each sample was hybridized to a custom designed probe set. Recovery and washing of captured targets were performed using the SeqCap hybridization and wash kit. The captured DNA targets were amplified using the KAPA HiFi HotStart ReadyMix. The amplified target-captured libraries were sequenced on an Illumina HiSeq 4000 System utilizing a patterned flow cell technology.

### 3.2. RNA Library Construction

One hundred nanograms of RNA per tumor sample was fragmented with heat in the presence of magnesium to an average size of 200 base pairs. The RNA then underwent first-strand cDNA synthesis using random primers, followed by combined second-strand synthesis and A-tailing, adapter ligation, bead-based cleanup, and library amplification. After library preparation, samples were hybridized with the IDT xGEN Exome Research Panel. Target recovery was performed using Streptavidin-coated beads, followed by amplification using the KAPA HiFi Library Amplification Kit. The RNA libraries were sequenced on an Illumina HiSeq 4000 System utilizing patterned flow cell technology to obtain approximately 65 million reads.

### 3.3. Radiomics Technique

Radiomics converted the tumor voxels into a minable dataset. All the provided T1-post DICOM 3D MRI images were converted to nrrd file format, and all lesions were contoured by radiologists. The lesion contours with its corresponding MRI images were resampled to 1 mm × 1 mm × 1 mm voxel resolution using B-spline interpolation and quantized into histogram bins for radiomics analysis. 1997 radiomic features were extracted from the verified lesion contours using the Tempus proprietary imaging platform. Features extracted from radiomics analysis can be roughly classified into 4 feature types: shape-based features (ex. 3D volume, 2D diameter, 3D diameter, and sphericity), intensity-based features (ex. energy, kurtosis, and skewness), texture-based features (ex. gray-level cooccurrence matrix (GLCM) and gray-level dependence matrix (GLDM)), and filter-based features (wavelet, log of sigma, etc.). Change in the value of radiomics features was used to track longitudinal changes in the tumor, such as the 3D tumor volume.

### 3.4. Digital Pathology Analysis

A tissue sample was stained with hematoxylin and eosin, then scanned using Philips Ultra-Fast Scanner at 40x resolution. The scanned 40x tiff H&E slide was reviewed and annotated by a pathologist. The pathologist annotated a majority of the areas that were pituitary adenoma and clear cell RCC (renal cell carcinoma). Tempus performed a deep-learning-based, pixel-level cell segmentation on the regions annotated by the pathologist to highlight the tumor cells. Based on the cell segmentation results and the labels of annotations where the cells were located, we could get the number and percentage of renal cells and pituitary cells, as well as the ratio of number of pituitary cells over number of renal cells. Microdissection of the tumor and deep-learning editing allowed for a separate analysis of the two tumors distinct pathology.

### 3.5. Literature Review

A systematic review was performed using PubMed search engine with such terms as “metastasis,” “pituitary adenoma,” “composite tumors,” and “collision tumors” to identify case reports of composite tumors in the sellar region of metastases and pituitary adenomas. All references from the reported cases were utilized to identify further case reports, resulting in the largest known collection of reported cases of metastatic lesions metastasizing into pituitary adenomas.

## 4. Discussion

Over the last 38 years, 34 cases of pituitary adenomas colliding with pituitary metastatic lesions have been reported (Table 1). Similar to our case report, in most of these cases, patients were radiographically diagnosed initially with pituitary adenomas only for pathologic studies to diagnose a mixture of pituitary adenoma and metastases. In the literature, the median patient age was 65.5 with 56.3% being females. The most common metastatic lesions originated from the lung and kidney with 10 and 6 cases, respectively. Regarding the pituitary tumor, 13 cases reported a nonfunctioning pituitary adenoma while 7 cases reported a prolactinoma.

Clinically, 22 patients presented with vision loss or ophthalmoplegia as the initial indication for surgery. Six cases involved hormonal abnormalities including Cushing's disease, acromegaly, and hypopituitarism. 27 patients reported undergoing surgery for the pituitary lesion. Of the 16 cases that reported postoperative mortality, the survival ranged from 0 to 24 months with a median of 3.5 months and a mean of 4.7 months. Of the 27 cases that commented on radiation therapy, 13 patients underwent radiation treatment.

Although rarely reported, autopsy evidence demonstrates that the sella hosts metastases in up to 27% of patients diagnosed with metastatic malignancy [35]. Meta-analysis studies described pituitary adenomas occurring in about 17% of the general population [36]. If there is no correlation or association with the tumors, then the probability of a patient who perishes from metastatic malignancy has a 5% chance of possessing a composite tumor consisting of a pituitary adenoma and metastases. However, with only 34 cases known the literature, the data does not align well. It remains unknown if pituitary adenomas influence metastases to the sella either positively, negatively, or not at all. One possible explanation is that the rich vascular network found in pituitary adenomas exposes the tumor to metastatic cells. Future clinicians should be weary that sellar masses in patients with history of metastatic cancer may appear similar to benign pituitary lesion but must be followed aggressively.

Regarding our patient's case, a few differential diagnostic possibilities should be considered based on microscopic findings. Pituitary adenomas and, in general, endocrine neoplasms can have extensive atypia and can undergo infarct, creating a malignant appearance, although these changes do not correlate with the biologic behavior or indicate pituitary carcinoma. Metastatic carcinomas from other sites, such as breast and lung, should also be considered in the differential diagnosis of the carcinoma component. Together with the characteristic cytohistomorphologic findings, immunohistochemical markers make this differential relatively straightforward. In our case, the patient's previously established diagnosis of renal cell carcinoma was also very helpful, even though the slides of the previous nephrectomy specimen, which was performed long ago at an outside institution, were not available for review. The presence of a lung lesion could also represent a second primary; however, microscopic and immunohistochemical findings did not support a lung origin. Meningioma may also be a consideration as part of a sellar collision tumor with pituitary adenoma [19, 37], and a clear cell meningioma may resemble clear cell renal cell carcinoma. Again, immunohistochemical findings strongly supported a renal cell carcinoma.

Genetic analysis revealed that the pituitary adenoma and the renal clear cell carcinoma shared a common *VHL* variant. Both the pituitary adenoma and the identified clear cell renal carcinoma expressed *VHL* P86S missense mutation leading to a loss of function. While the pituitary adenoma had a VAF of 4.8%, the identified clear cell renal carcinoma had a VAF of 24.4%. Similar mutations have been documented in cases of renal cell carcinoma [38, 39]; however, recent studies have reported *VHL* inactivation alone is insufficient to cause tumor formation [40]. We do not have germ cell data to ascertain if our patient had VHL disease; however, the presence of VHL and NOTCH2 in both tissue samples and the history or renal clear cell carcinoma suggest an abnormality that points towards germ-line mutation. Our patient's tumor had a stop mutation in the *PBRM1* gene which is the second most commonly mutated gene in RCC and is associated with normal cell proliferation. Additional mutations of relevance were *NOTCH2* copy number loss in both the renal cell carcinoma and pituitary adenoma which could lead to impaired regulation [41], an *NF1* splice variant in the RCC which could affect normal cell proliferation [42]. While there was an apparent *STAG2* stop mutation in the RCC that could lead to impaired division, most *STAG2* mutations are associated with bladder cancer [43], but there is little genetic analysis on the association of *STAG2* to RCC. This is distinct from the most common genetic deficiencies found for pituitary adenomas, the most common genetic mutations being loss of function of *MEN1* [44], Gs-alpha protein activation [45], and *AIP* gene in familial isolated pituitary adenoma [4]. While the genotypic analysis had little effect on the diagnosis and prognosis of the patient, it is the first instance of tpdelmolecular analysis of composite tumors; further sequencing can improve our understanding of tumor metastasis mechanisms.

In conclusion, composite tumors of the pituitary region are more morbid than pituitary adenomas. Treatment should be guided based on the most aggressive tumor, and adjuvant radiation should be used most often. Next-generation sequencing data can be successfully carried out on each component of tumor and be used as an avenue to identify potential targets as well as potentially understand pathogenesis [46]. We hope other clinicians in the future will consider genomic analysis to better understand the genetic cause of composite tumors and improve treatment modalities.

## Figures and Tables

**Table 1:** Literature review of clinical cases involving metastasis of systemic malignancy into a pituitary adenoma.

Cancer		Age	Gender	Pituitary tumor	Presentation	Radiation?	Post-op survival
Lung cancer	Sogani et al. 2014 [6]	64	M	Nonfunctioning	Vision loss	Yes	Not reported
	Hoellig et al. 2009 [7]	71	M	Nonfunctioning	Vision loss	No	13 days
	Rotondo et al. 2013 [8]	66	M	Prolactin	Headache	No	No surgery
	Molinatti et al. 1985 [9]	71	M	LH/FSH	Vision loss	Yes	1 month
	Post et al. 1988 [10]	77	M	Nonfunctioning	Vision loss	Yes	6 months
	Kovacs, 1973 [11]	N/A	N/A	N/A	N/A	N/A	N/A
	Scheithauer, 1984 [12]	N/A	N/A	N/A	N/A	N/A	N/A
	Hanna et al. 1999 [13]	42	F	Prolactin	Headache	Not reported	Not reported
	Nasr et al. 2006 [14]	44	F	Growth hormone	Vision loss/acromegaly	Yes	>3 months
	Fujimori et al. 2014 [15]	80	M	Not reported	Vision loss/CN 3 palsies	Yes	Not reported
Renal cancer	James et al. 1984 [16]	75	M	Nonfunctioning	Vision loss	Yes	Not reported
	Koutourousiou et al. 2010 [17]	42	F	ACTH	Cushing's syndrome	Not reported	>55 months
	Koutourousiou et al. 2010 [17]	76	M	Nonfunctioning	Panhypopituitarism	Not reported	>27 months
	Weber et al. 2003 [18]	62	F	Nonfunctioning	Vision loss	Not reported	8 months
	Burns et al. 1973 [19]	78	M	Nonfunctioning	Fever	Not reported	No surgery
	Magnoli et al. 2014 [20]	76	F	FSH/LH	Vision loss, CN 3 palsies	No	24 months
Breast cancer	Bret et al. 2001 [21]	75	F	FSH/LH	Vision loss	No	>18 months
	Richardson and Katayama, 1971 [22]	70	F	Nonfunctioning	Headache/L hemiparesis	Yes	4 months
	van der Zwan et al. 1971 [23]	73	F	Nonfunctioning	Vision loss	No	1 month
	Zager et al. 1987 [24]	56	F	Nonfunctioning	CN 6 palsies	Yes	No surgery
	Mills et al. 2018 [25]	65	F	FSH/LH	Apoplexy	No	12 days
Idiopathic	Nassiri et al. 2012 [26]	55	F	Growth hormone	Acromegaly	Yes	5 months
	Bret et al. 2001 [21]	87	F	FSH/LH	Vision loss	No	>6 months
	Hurley et al. 1992 [27]	76	M	Growth hormone	Vision loss/acromegaly	No	6 months
	Post et al. 1988 [10]	61	F	ACTH	Vision loss	Yes	Not reported
Colon	Noga et al. 2001 [28]	60	M	Nonfunctioning	Vision loss	No	“Shortly”
	Skulsampaopol et al. 2017 [29]	53	M	Nonfunctioning	Vision loss	Yes	3 months
	Thewjitcharoen et al. 2014 [30]	65	M	Prolactin	Vision loss	No	9 months
Stomach	Van Seters et al. 1985 [31]	66	F	Prolactin	Vision loss	No	12 days
	Molinatti et al. 1985 [9]	66	F	Prolactin/growth hormone	Vision loss	No	2 days
Prostate	Ramsay et al. 1988 [32]	57	M	Nonfunctioning	Incidental	Yes	No surgery
Pancreas	Ramsay et al. 1988 [32]	50	F	ACTH	Cushing's syndrome	Yes	No surgery
Mediastinum	Abe et al. 1997 [33]	46	F	Prolactin	Vision loss	No	6 months
Melanoma	Yang et al. 2017 [34]	62	F	Prolactin	Vision loss	No	>22 months

## Data Availability

N/A.
